# Effects of system tuning and RAM disk on the performance of a clinical laboratory information system

**DOI:** 10.1155/S146392468900026X

**Published:** 1989

**Authors:** Arthur A. Eggert, Kenneth A. Emmerich, Thomas J. Blankenheim, Gary J. Smulka

**Affiliations:** 1 Department of Pathology and Laboratory Medicine University of Wisconsin Madison Wisconsin 53792 USA; 2 Clinical Laboratories University of Wisconsin Hospital Madison Wisconsin 53792 USA

## Abstract

Improvements in the performance of a laboratory computer system do not necessarily require the replacement of major portions of the system and may not require the acquisition of any hardware at all. Major bottlenecks may exist in the ways that the operating system manages its resources and the algorithm used for timesharing decisions. Moreover, significant throughput improvements may be attainable by switching to a faster storage device if substantial disk activity is performed. In this study the fractions of time used for each of the types of tasks a laboratory computer system performs (e.g. applications programs, disk transfer, queue cycler) are defined and measured. Methods for reducing the time fractions of the various types of overhead are evaluated by doing before and after studies. The combined results of the three studies indicated that a 50% improvement could be gained through system tuning and faster storage without replacement of the computer itself


Journal of Automatic Chemistry Vol. 11, No. 3 (May–June 1989), pp. 119–123

### Effects of system tuning and RAM disk on the performance of a clinical laboratory information system

**Arthur A. Eggert**

*Department of Pathology and Laboratory Medicine, University of Wisconsin, Madison, Wisconsin 53792, USA*

**Kenneth A. Emmerich, Thomas J. Blankenheim and Gary J. Smulka**

*Clinical Laboratories, University of Wisconsin Hospital, Madison, Wisconsin 53792, USA*

*Improvements in the performance of a laboratory computer system do not necessarily require the replacement of major portions of the system and may not require the acquisition of any hardware at all. Major bottlenecks may exist in the ways that the operating system manages its resources and the algorithm used for timesharing decisions. Moreover, significant throughput improvements may be attainable by switching to a faster storage device if substantial disk activity is performed. In this study the fractions of time used for each of the types of tasks a laboratory computer system performs (e.g. applications programs, disk transfer, queue cycler) are defined and measured. Methods for reducing the time fractions of the various types of overhead are evaluated by doing before and after studies. The combined results of the three studies indicated that a 50% improvement could be gained through system tuning and faster storage without replacement of the computer itself.*

### Introduction

The performance of any computer system is limited by both the speed and versatility of its hardware and the efficiency of its software. Historically, computer systems have been purchased because they meet the current requirements and are projected to meet the future requirements for 2–5 years. Unfortunately, the usefulness of computer technology is so great that computers are usually saturated in less than half of the time projected. This leads to substantial hardware replacement expense and frequently means that major software systems must also be replaced, redeveloped or at least reimplemented on new hardware. The accompanying disruption of service reduces the value of computer support to an analytical laboratory. Evaluating methods for reducing the need for major hardware replacements is therefore essential not only to reduce costs, but also to prevent service disruptions that are encountered when actual system replacement occurs.

Understanding why laboratory computer systems become obsolete is the first step in determining how to delay the onset of such obsolescence. Four major areas of concern can be readily identified, viz. storage capacity, hardware speed, operating system efficiency and application software efficiency. The first of these areas of concern can usually be addressed without system replacement by the addition of large storage devices and is therefore a solved problem. The last area is one which is generally in the hands of applications programmers. There is no good method of rating efficiency except through benchmarking the programs against specific tasks. Even this seldom indicates what the bottlenecks are in a piece of software currently in use. As a consequence, only hardware speed and operating system efficiency are left as topics to study for someone who wants to improve the performance and therefore extend the lifetime of current hardware systems.

As both of the phenomena of interest involve the amount of time the system needs to accomplish a task, it is necessary to break the system time into its components. In a previous paper [1] we indicated that

$$\text{system time} = \text{program time} + \text{queue cycler time} + \text{disk wait time}$$

In fact, as the demands on a system increase, it may be necessary to permit more users on to the system than there are memory partitions or space to hold the application programs which are active. This means that users are 'swapped out,' that is, their programs are transferred to a disk buffer while some other user takes over their memory partitions. Part of the time needed to swap is a result of the disk speed and part is a result of the efficiency of the swapping algorithm. Collectively these times can be designated as 'swap time' and must be added to the expression above to give a complete equation:

$$\text{system time} = \text{program time} + \text{queue cycler time} + \text{disk wait time} + \text{swap time}$$

The important point to grasp about the system time equation is that it is really an equation in terms of percentages and not absolute numbers. If a computer system goes faster, there is effectively more system time per every unit of clock (real) time. Traditional benchmarking studies effectively show how much system time occurs in how much real time, but this is not a useful approach for these studies, as we are not looking at whether to change systems, but at how the lifetime of a system can be extended. What we need to accomplish, therefore, is to maximize the program time by pushing it as close as possible to 100% of the system time [2]. This paper reports on our attempts to accomplish this through studying how to minimize the other three terms in the equation.

### Methods and materials

The Clinical Laboratories of the University of Wisconsin Hospital and Clinics run a RelationaLABCOM (LAB-

COM+) system from Laboratory Consulting Inc. (LCI), of Madison, WI, USA. LABCOM+ is a self-contained operating system/database management package, but it is general enough that the conclusions drawn should be applicable to other operating system environments [3]. The software runs on a PDP 11/84 with 4 megabytes of main memory [Digital Equipment Corp. (DEC), Maynard, MA, USA], 336 megabytes of moving head disk (an RL-02, an RA-80 and an RA-60) and 4 megabytes of RAM disk (16 more were added as part of this study) from Imperial Technology Inc. A wide variety of terminals are present, including several varieties of teleprinters, several types of video display terminals (including both formatted and teleprinter equivalent) and several IBM-XT-compatible personal computers. There are two Southern Systems QT 600 line printers, a Hewlett-Packard 7260A card reader, a DEC TS11 tape drive and numerous character printers. The system has interfaces to the central hospital computer running ACTION 2000 by SMS, to a DECnet with two PDP 11/44s in the Clinical Laboratories running RSX 11M+ and to numerous laboratory instruments.

*Reducing swap time*

A well known computer system problem is that after a certain number of users have been added to a time-sharing system, the increase in response time begins to grow exponentially rather than linearly with each user added. A major contribution to this growth is the necessity for the system to remove temporarily some active users from memory to disk to permit other users to have enough memory resources to run. Many schemes have been devised to make this efficient by swapping out user programs which are waiting for something to happen before they can resume execution and therefore cannot be running anyway. Even the best swapping scheme can fail, however, if the assumptions made when it was developed are not true because the system operating conditions are not those predicted by the designers. Figure 1 shows the percentage of system time that was used for swapping over the course of the first shift of our laboratory.

It is obvious from figure 1 when the peak activities in the laboratory occurred, yet it was also clear to the authors that the peaks in the figure were much higher relative to the baseline than the actual system usage peaks. Examination of the source code showed us the reason for the problem. The swap algorithm caused the last active program to be swapped out when there was not enough space to hold all the programs that users had called. This algorithm would have worked well if all programs were equally active, as the program that would be accessed last in the group of programs to be executed would be removed from memory. Unfortunately, this is not how a clinical laboratory system really operates. Many users will log on to the system and call programs that are used only intermittently during the day. On the other hand, printing programs, once activated, search and format intensively and continuously until completed. Therefore, once the memory had filled up with semi-inactive programs, if two or more printing programs were running at the same time they would continually be swapped in and out while the large number of semi-active programs would remain continually in memory even though they rarely ran. In other words, when the system became busy, it stood in its own way, making it even busier. To eliminate this problem, a pointer was established to the last program swapped out. Whenever another program needs to be swapped, the cycler begins at the pointer to work its way aroung the queue looking for programs which are waiting on external events (e.g. user input, device availability) and swaps out the first program found which is still in memory. As a consequence, the programs that are eligible to run at the next available opportunity are left in memory, and those which are less likely to run are removed. This is not foolproof because it is possible that a program whose run conditions are about to be met will occasionally be transferred to disk; nevertheless, after several passes around the queue, the group of swapped-out programs is composed almost exclusively of those which are effectively inactive. When suitable changes to the source code were made to implement this new strategy, swapping became rare, as shown in figure 2.

*Reducing queue cycle time*

To manage a timesharing system one must have a queue cycler. The queue cycler divides the resources of the system based on some sort of fairness and priority scheme. In LABCOM+, as in most timesharing systems, there are definite levels of priority such that all those things at a higher level of priority always run in

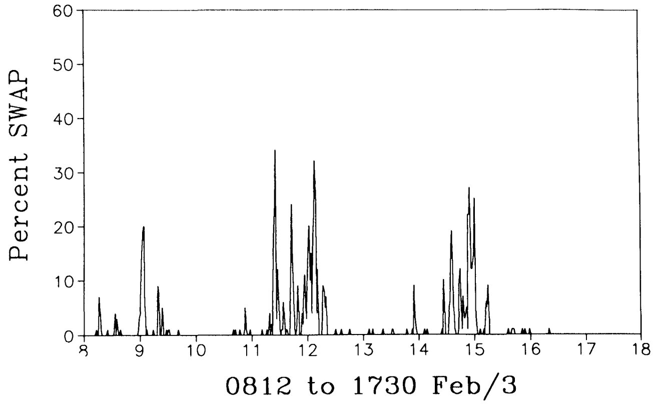

*Figure 1. The swap time is excessive during times of heavy use when the criterion for swapping is 'swap out last program run.'*

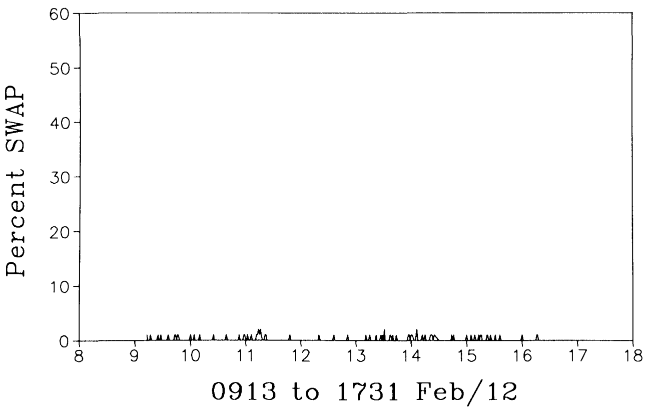

*Figure 2. The swap time is almost non-existent when the criterion for swapping is 'swap out next program which is probably inactive.'*

preference to those things at a lower level. In LAB-COM+, the interfaces run at a higher priority than the interactive programs, which run at a higher priority than the printing programs. This means that the printing programs only get the crumbs of time that are available and cannot afford to waste any time when given them. On the other hand, because clinical laboratory systems must allow multiple users to access the same patient file at the same time, they must be very careful in what state the files are left when they switch between programs. Therefore, while many timesharing systems use a rigid 'time slice' algorithm where programs are always switched after a certain fraction of a second, this is not always practical in clinical laboratory systems. LABCOM+ bases its decision to switch programs on how many disk transfers have been done since the program was initiated.

There are two difficulties which have been detected with the approach of counting disk transfers. The first is that not all programs have the same computation to disk transfer ratio. Search programs rapidly use up their disk transfer allotments while computation programs use them slowly, thereby receiving much more generous fractions of computer time. As printing programs tend to be search intensive, they get only a small amount of time while, as mentioned above, running at the lowest priority. Secondly, information is stored on different types of mass storage devices, each of which can have a different response time. Programs that read from short response time storage can do the same number of transfers in much less time than those that must rely on long response time storage.

The inability of programs to accomplish much because they do not get enough real time as a result of the method of time sharing employed shows up as an excessive amount of time spent in the queue cycler (figure 3). This occurs because the queue cycler uses a fixed amount of time whenever it must transfer between programs. If it has to transfer often because programs are using very little time, then the queue cycler time will be high. As queue cycler time is pure overhead, it reduces the throughput of the system. Increasing the number of disk transfers before a swap does not solve this problem. It will indeed decrease the percentage of queue cycler time, but it also means that those programs which do few disk

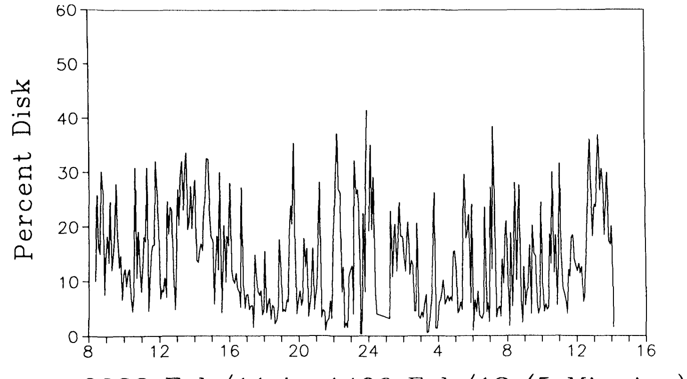

*Figure 3. The queue cycler uses a large fraction of the time when programs are switched too frequently.*

transfers will remain resident for such long intervals that system users will notice a distinct increase in response time. More evenness can be obtained by making the number of transfers dependent on the speed of the transfer device used, giving proportionally more transfers to faster devices than to slower devices. This is not always simple to implement, and it does not solve the whole problem. What is needed is some way to hold programs that do a large number of disk transfers in memory longer relative to those which compute more, while taking into consideration that many programs change back and forth between heavy computation and heavy information transfers.

The method we developed after some study was the WAIT SLICE. Whenever a program is restarted by the queue cycler, the time is saved. When the preset number of disk transfers is reached, the cycler checks the clock to see if a preset number of clock ticks (sixty per second) has elapsed. If not, the disk transfer counter is cleared, and the program is permitted to keep running. If the time limit is exceeded, then the program is suspended and the next program started. In effect, this guarantees that each program will never be shortchanged on time, no matter how it uses it. Figure 4 shows the reduction of queue cycler overhead due to this new way of deciding when to change active programs.

*Mass storage speed*

Improving hardware speed invariably means purchasing new equipment. Which piece of equipment to purchase, however, might not be obvious. Frequently one can gain significant performance improvement by replacing only a small part of the overall system. The problem is to identify the appropriate part. If the percentage of system time spent in doing disk transfers is significant, then improvement can be obtained through the use of a faster storage device. Figure 5 shows a configuration where only volatile files (those which are not essential to start the system in case of power failure) such as scratch files and on-line data storage were kept on 4 megabytes of RAM disk storage (RAM disk is random access memory which is set up to be handled like a disk by the computer). Most of the patient files were kept on moving head disk (an RA-80) with an average retrieval (i.e. access plus transfer) time of 27 ms. As an experiment, we configured most of the heavily used parts of these files on to another 16 megabytes of RAM disk (retrieval time of 2 ms). The amount of disk wait time dropped dramatically, as can be seen in figure 6. This confirms what was demonstrated in our previous paper on hardware improvements, namely that the speed of data storage has a noticeable effect on the performance of a clinical laboratory information system.

### Results and discussion

The nature of research into computer system performance is always such that everything that one tries that does not work is insignificant once one discovers what does, and what does work is always so obvious with hindsight that the data justifying it seem superfluous. The major thrust behind our efforts continues to be to define

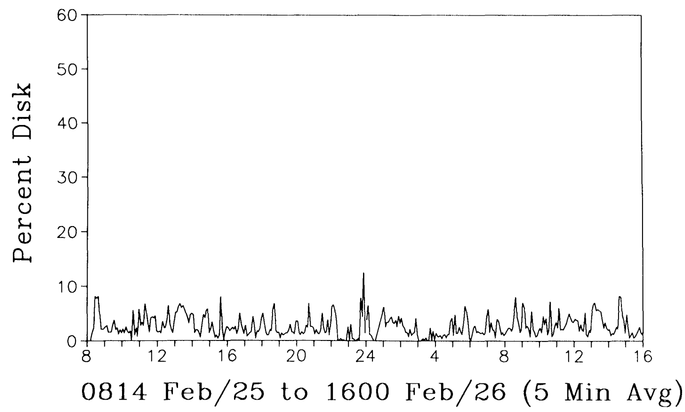

*Figure 4. The queue cycler uses less time and its proportion of time used is less erratic when time slices are made more even.*

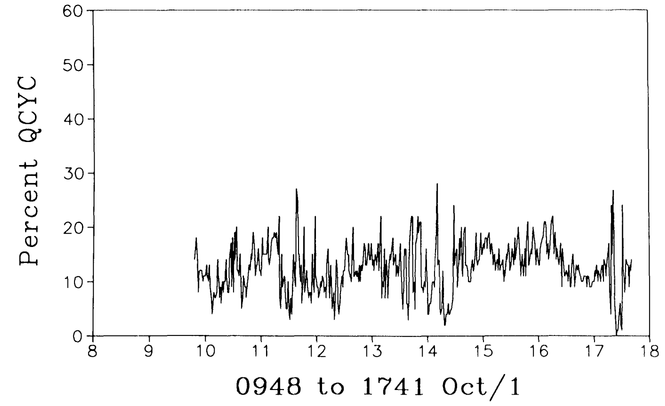

*Figure 5. Using a conventional moving-head disk for patient data storage causes numerous high peaks of wasted time waiting for disk activity.*

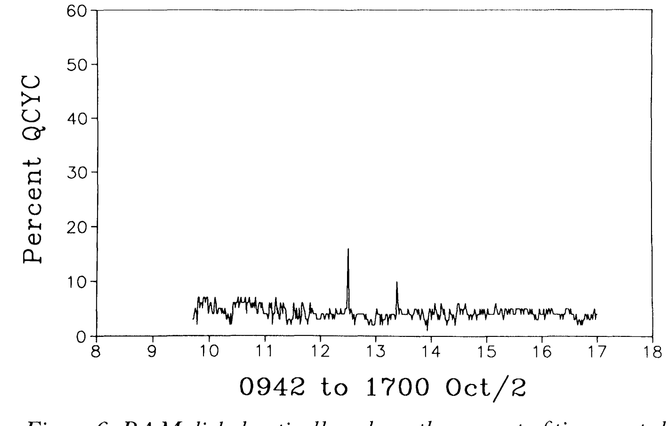

*Figure 6. RAM disk drastically reduces the amount of time wasted for the completion of disk transfers.*

'system time' in terms of its components so precisely that anyone with access to a resource monitor can determine what the bottlenecks to the performance in his system are and what type of actions must be taken to remove them. Although we have dramatically improved the throughput of our own system (and therefore that of many Relationa-LABCOM users) over the last few years, we sense a need for all chemistry laboratories, clinical and otherwise, to have tools to diagnose their own computer system performance and to be able to use these tools to tune these systems to minimize the capital outlay for new equipment.

The swap time study illustrates the importance of graphing activity over the whole time frame rather than just looking at system averages. The average swap time was initially only 1·7% of the system time, which seems to be so small that it would not be worth trying to reduce it. Although we were able to reduce it to nearly zero (0·1%), this could be argued as being less than a 2% improvement (99·9% versus 98·3%) in overall system performance. The time graph shows a different story. The peak swap time reached 34·0% of system time, and this peak swap time occurred when the system was under maximum use. At such times a loss of one third of the system's capacity was indeed significant. The maximum swap time was reduced to 2·0%, which was a 48% improvement (98% versus 66%) in the time available. Identifying where the time is being spent during peak periods is therefore significant. We considered an alternative swapping criterion, namely swapping out the program that had not been used for the longest period of time, but this would have required some alterations to the system files to permit the storage of this information. Because there was so little additional gain to be attained even if this latter scheme were more effective, no experimentation with it was done.

The queue cycler study was the most difficult because there was no obvious way to proceed. The queue cycler strategy was a result of the basic system design and it was not possible to change it. On the other hand, any overhead function which is consuming an average of 12·4% and a peak of 28·0% of the available time is not an ignorable problem. Although the implementation of the WAIT SLICE reduced the average time to 4·2% and the maximum to 16% of the system time, it is not a perfect solution because it does not completely even out the time queues. If a program has just barely missed using its maximum time, it will be allowed another whole disk transfer cycle, even though this will carry it well past the target average. Although this is undesirable, the calculation necessary at each potential exit is also expensive in terms of time owing to the layering of the operating system. A balance point we chose was one which slightly degraded the response time to prevent expending more calculation time, which is an overhead.

Finally, with the installation of the RAM disk the average system time spent in disk activity dropped from 14·4 to 2·7%, with the peak falling from 51·0 to 20·0%. This change also enhanced the gain made from installing the WAIT SLICE, as the queue cycler time fell to 3·8%. The total impact of these three improvements was to increase the program time at full load from approximately 57 to 88% of the system time, that is, by more than 50%. Such performance enhancement is noticeable by system users who now see few periods in which response time or throughput are unacceptable. With these studies, however, we have nearly exhausted methods for reclaiming program time. Even if we were to force everything but program time to 0%, we would gain only 15% more performance. In fact, reclaiming about a third of the remaining time is probably the best we can do. Major performance improvements in the future can only come from better written applications programs (a doubtful source) and offloading some of the work to microcomputers. Nevertheless, we feel that we have conclusively demonstrated that substantial performance enhancements can be made in many laboratory computer systems by using resource monitors to identify the bottlenecks and then making the relatively inexpensive corrections to remove them.

### References

1. Eggert, A. A., Smulka, G. J., Blankenheim, T. J. and Emmerich, K. E., *Journal of Automatic Chemistry*, **9** (1986), 37.
2. Shaw, A. C., *The Logical Design of Operating Systems*, (Prentice-Hall: Englewood Cliffs, NJ, 1974), pp. 198–202.
3. *RelationaLABCOM User Manual* (Laboratory Consulting: Madison, WI, 1988).

### NOTES FOR AUTHORS

*Journal of Automatic Chemistry* covers all aspects of automation and mechanization in analytical, clinical and industrial environments. The *Journal* publishes original research papers; short communications on innovations, techniques and instrumentation, or current research in progress; reports on recent commercial developments; and meeting reports, book reviews and information on forthcoming events. All research papers are refereed.

### Manuscripts

Two copies of articles should be submitted. All articles should be typed in double spacing with ample margins, on one side of the paper only. The following items should be sent: (1) a title-page including a brief and informative title, avoiding the word 'new' and its synonyms; a full list of authors with their affiliations and full addresses; (2) an abstract of about 250 words; (3) the main text; (4) appendices (if any); (5) references; (6) tables, each table on a separate sheet and accompanied by a caption; (7) illustrations (diagrams, drawings and photographs) numbered in a single sequence from 1 upwards and with the author's name on the back of every illustration; captions to illustrations should be typed on a separate sheet. Papers are accepted for publication on condition that they have been submitted only to this *Journal.*

### References

References should be indicated in the text by numbers following the author's name, i.e. Skeggs [6]. In the reference section they should be arranged thus:

to a journal

Manks, D. P., *Journal of Automatic Chemistry*, **3** (1981), 119.

to a book

Malmstadt, H. V., in *Topics in Automatic Chemistry*, Ed. Stockwell, P. B. and Foreman, J. K. (Horwood, Chichester, 1978), p. 68.

### Illustrations

Original copies of diagrams and drawings should be supplied, and should be drawn to be suitable for reduction to the page or column width of the *Journal*, i.e. to 85 mm or 179 mm, with special attention to lettering size. Photographs may be sent as glossy prints or as negatives.

### Proofs and offprints

The principal or corresponding author will be sent proofs for checking and will receive 50 offprints free of charge. Additional offprints may be ordered on a form which accompanies the proofs.

*Manuscripts should be sent to Dr P. B. Stockwell, P.S. Analytical Ltd, Arthur House, Unit B4, Chaucer Business Park, Watery Lane, Kemsing, Sevenoaks, Kent TN15 6QY, UK.*

